# Effects of passive leg raising and volume expansion on mean systemic pressure and venous return in shock in humans

**DOI:** 10.1186/s13054-015-1115-2

**Published:** 2015-11-23

**Authors:** Laurent Guérin, Jean-Louis Teboul, Romain Persichini, Martin Dres, Christian Richard, Xavier Monnet

**Affiliations:** AP-HP, Hôpitaux universitaires Paris-Sud, Hôpital de Bicêtre, service de réanimation médicale, 78, rue du Général Leclerc, Le Kremlin-Bicêtre, F-94270 France; Univ Paris-Sud, Faculté de médecine Paris-Sud, Inserm UMR_S 999, Le Kremlin-Bicêtre, F-94270 France

## Abstract

**Introduction:**

The aim of this study was to assess how mean systemic pressure (Psm) and resistance to venous return (Rvr) behave during passive leg raising (PLR) in cases of fluid responsiveness and fluid unresponsiveness.

**Method:**

In 30 patients with an acute circulatory failure, in order to estimate the venous return curve, we constructed the regression line between pairs of cardiac index (CI) and central venous pressure (CVP). Values were measured during end-inspiratory and end-expiratory ventilatory occlusions performed at two levels of positive end-expiratory pressure. The x-axis intercept was used to estimate Psm and the inverse of the slope to quantify Rvr. These measurements were obtained at baseline, during PLR and after fluid infusion. Patients in whom fluid infusion increased CI by more than 15 % were defined as “fluid-responders”.

**Results:**

In fluid-responders (n = 15), CVP and Psm significantly increased (from 7 ± 3 to 9 ± 4 mmHg and from 25 ± 13 to 31 ± 13 mmHg, respectively) during PLR. The Psm-CVP gradient significantly increased by 20 ± 30 % while Rvr did not change significantly during PLR. In fluid-nonresponders, CVP and Psm increased significantly but the Psm-CVP gradient did not change significantly during PLR. PLR did not change the intra-abdominal pressure in the whole population (14 ± 6 mmHg before vs. 13 ± 5 mmHg during PLR, p = 0.26) and in patients with intra-abdominal hypertension at baseline (17 ± 4 mmHg before vs. 16 ± 4 mmHg during PLR, p = 0.14). In the latter group, PLR increased Psm from 22 ± 11 to 27 ± 10 mmHg (p <0.01) and did not change Rvr (5.1 ± 2.6 to 5.2 ± 3 mmHg/min/m^2^/mL, p = 0.71). In fluid-responders, Psm, CVP and the Psm-CVP gradient significantly increased during fluid infusion while the Rvr did not change. In fluid-nonresponders, CVP and Psm increased significantly during fluid infusion while the Psm-CVP gradient and Rvr did not change.

**Conclusion:**

PLR significantly increased Psm without modifying Rvr. This was also the case in patients with intra-abdominal hypertension. In case of fluid responsiveness, PLR increased venous return by increasing Psm to a larger extent than CVP. In patients with fluid unresponsiveness, PLR increased Psm but did not change the Psm–CVP gradient. Fluid infusion induced similar effects on Psm and Rvr.

## Introduction

According to the Guyton model of circulation, systemic venous return is determined by two components [1]. The first is the pressure gradient between the mean systemic pressure (Psm) and the right atrial pressure, which tends to promote venous return. The second is the resistance to venous return (Rvr), which tends to impede venous return [1].

Passive leg raising (PLR) has been developed as a test to predict fluid responsiveness. This postural manoeuvre is supposed to transfer a significant volume of venous blood toward the intrathoracic compartment [2]. However, it has been suggested that PLR could have non-significant effects on cardiac preload [3], in particular in the case of intra-abdominal hypertension [4]. This would result in a negative PLR test in spite of an actual fluid responsiveness. In the present study conducted in patients with acute circulatory failure, we aimed to assess how Psm and Rvr behave during PLR in cases of fluid responsiveness and fluid unresponsiveness. In particular, we aimed to investigate whether the absence of increase in cardiac output during PLR is due to an absence of increase in Psm, resulting in the absence of a significant increase in cardiac preload, or to a preload independence *per se*, that is to say, to an absence of increase in cardiac output to a significant increase in cardiac preload.

Estimating Psm and Rvr at the bedside has been difficult for many years, as it requires measurement of intravascular systemic pressure during cardiac arrest [5, 6]. Recently, an elegant method has been proposed by Maas and co-workers to estimate Psm and Rvr at the bedside [7]. This method is based on the recording of several pairs of measurements of cardiac output and central venous pressure (CVP) obtained by varying the intrathoracic pressure. Our group modified this method in order to widen the range of values of these pairs of measurements [8]. In the present study, we used this method to investigate the effects of PLR on Psm and Rvr.

## Methods

### Patients

The study was performed in the medical intensive care unit of a University Hospital. It was approved by the Institutional Review Board of our institution (Comité pour la protection des personnes Ile-de-France VII). Deferred informed consent was requested from the patient’s surrogate as soon as possible. As the patient recovered consciousness, deferred informed consent was requested from the patient. If the patient or his/her next of kin refused to consent, the patient’s data were not entered into the analysis. Patients were included in the study if they met all the following criteria:

1. Decision of the attending physician to perform a PLR test and fluid infusion
2. Age ≥18 years
3. Mechanical ventilation in the volume assist control mode (Evita 2 or 4, Dräger, Lübeck, Germany)
4. State of consciousness allowing 15-sec expiratory and inspiratory occlusions to be performed, as assessed by visual observation of the airway pressure curve displayed by the ventilator

Patients were excluded if PLR was contraindicated (intracranial hypertension, venous compression stocking).

### Haemodynamic measurements

All patients had an internal jugular vein catheter and a thermistor-tipped arterial catheter (PV2024, Pulsion Maquet, Munich, Germany) in the femoral artery. The pressure sensors connected to the arterial and venous lines were referenced to the right atrium, corresponding to the axillary line, 5 cm below the sternal angle and zeroing was performed against atmospheric pressure. Arterial pressure, CVP and intra-abdominal pressure were continuously recorded by using data acquisition software (HEM 4.2, Notocord, Croissy-sur-Seine, France). The beat-to-beat values of stroke volume derived from pulse contour analysis performed by the PiCCO2 device were computerised by using the PiCCOWin 4.0 software (Pulsion Maquet). These beat-to-beat values of stroke volume were then averaged over a 2-sec period and cardiac index (CI) was calculated over this period. Calibration of pulse contour analysis-derived estimation of stroke volume was performed by transpulmonary thermodilution with injection of three cold saline boluses (15 mL each) at baseline and after fluid infusion.

### Method used to estimate Psm and Rvr

The method used to estimate Psm and Rvr has been previously described [8]. It is based on the principle that the venous return curve is the relationship between right atrial pressure (abscissa) and venous return (ordinate). At steady state, the right atrial pressure could be estimated through CVP and the venous return through CI [7]. Venous return curves were constructed by obtaining a series of points with various CI and CVP values. For this purpose, CI and CVP were simultaneously recorded during 15-sec end-inspiratory and end-expiratory holds. Aiming at enlarging the range of CI and CVP values, we performed occlusions at two different levels of positive end-expiratory pressure (PEEP): first, at PEEP = 5 cmH_2_O, then at PEEP set for reaching a plateau pressure of 28–30 cmH_2_O [8].

During each respiratory hold, we recorded the extreme values of CI (averaged over 2 sec) and the value of CVP at the same time. A regression line was computed (least-squares method, Excel, Microsoft, Redmond, WA, USA) between the four pairs of measurements obtained at baseline, during PLR and after volume expansion. This regression line is assumed to equate the venous return line. The Psm was estimated as the pressure corresponding to the x-intercept of the regression line, as described in the model proposed by Guyton et al. [1]. Rvr was estimated from the inverse of the slope of the regression line.

### Measurement of intra-abdominal pressure

Intra-abdominal pressure was estimated by the bladder pressure after the injection of 25 mL of saline solution in the Foley catheter with the patient in the semi-recumbent position [9]. The abdominal pressure transducer was fixed to the patient on the lateral side of the pelvis, 2 cm below the anterior superior iliac spine and zeroing was performed against atmospheric pressure [10]. During PLR, it was carefully checked that the height of this transducer remained unchanged.

### Study design

At baseline, a first set of measurements was performed, including arterial pressure, CVP, intra-abdominal pressure and CI measured by transpulmonary thermodilution. Four respiratory holds, two at end-expiration and two at end-inspiration, were randomly performed as described above in order to estimate Psm and Rvr at baseline.

After performing these respiratory occlusions, when CI had returned to its baseline value, a PLR test was performed. For this purpose and as previously described [2, 11], the patient was moved from the semi-recumbent position to a position where the trunk was supine and the legs were lifted at 45°. After stabilisation of CI, i.e., within 1 minute, another series of four respiratory holds was repeated in order to estimate Psm and Rvr during PLR.

After these recordings, the patient was moved back to the semi-recumbent position and CI was allowed to stabilise. After stabilisation, fluid infusion was performed by infusing 500 mL saline over 10 minutes. After fluid infusion, a third series of respiratory holds was performed in order to estimate Psm and Rvr. All other treatments were kept unchanged during the study period.

### Data analysis

Date are expressed as mean ± SD or frequency (n, %). All quantitative data were normally distributed (Kolmogorov test). Comparison between the three time points of the study was performed using the paired Student’s *t* test with Bonferroni correction for repeated measurements. Patients in whom fluid infusion increased CI by more than 15 % were defined as fluid-responders. Comparison between fluid-responders and fluid-nonresponders was performed using the two-tailed Student’s *t* test. Comparison of proportions was performed with the chi-square test. A receiving operating characteristics (ROC) curve was constructed to assess the ability of the PLR-induced changes in CI to detect fluid responsiveness. The best cutoff value of PLR-induced changes in CI was defined as the one providing the best Youden index. Statistical significance was defined by a *p* value <0.05. The statistical analysis was performed using MedCalc 11.6.0 software (MedCalc, Mariakerke, Belgium).

## Results

### Patients’ characteristics

Thirty patients were included in the study. Their characteristics are summarised in Table 1. The most frequent aetiology of shock was sepsis. Sedation was administered in 29 patients and neuro-muscular blocking agents in 18 patients. Fluid infusion increased CI by more than 15 % in 15 fluid-responders.

**Table 1:** Patients characteristics at baseline

	Fluid responders	Fluid nonresponders	*P*
	n = 15	n = 15	
Age, years	62 ± 10	67 ± 13	0.25
Weight, kg	78 ± 22	78 ± 22	0.99
Height, cm	169 ± 10	168 ± 13	0.76
Shock aetiology			
Septic (n, %)	9 (60 %)	10 (67 %)	1,00
Cardiogenic (n, %)	4 (27 %)	4 (27 %)	1,00
Hypovolemic (n,%)	2 (13 %)	1 (6 %)	1,00
Simplified Acute Physiologic Score II	56 ± 20	60 ± 14	0.45
Male gender (n, %)	9 (60 %)	12 (80 %)	0.43
Tidal volume, mL/kg of ideal body weight	6.5 ± 1,0	6.6 ± 1,0	0.67
Patients receiving norepinephrine (n, %)	14 (94 %)	9 (60 %)	0.08
Dose of norepinephrine, ig/kg/min	0.40 ± 0,32	0.28 ± 0.34	0.31
Patients receiving dobutamine (n,%)	1 (6 %)	2 (13 %)	1,00
Dose of dobutamine, g/kg/min	0.67 ± 2.58	0.67 ± 1.76	1,00
Renal replacement therapy (n, %)	6 (40 %)	6 (40 %)	1,00
Patients receiving propofol (n,%)	14 (94 %)	15 (100 %)	1,00
Dose of propofol, mg/h	183 ± 99	207 ± 68	0.46
Patients receiving rem ifentanyl (n, %)	7 (47 %)	8 (53 %)	1
Dose ofremifentanyl, μg/h	113 ± 146	97 ± 130	0.74

Values are expressed as mean ± standard deviation or as n (%)

Considering the whole population at baseline, increased intra-abdominal pressure was present (equal to or higher than 12 mmHg) in 16 patients. It was of grade I (between 12 and 15 mmHg [9]) in seven patients, grade II (between 16 and 20 mmHg) in seven patients and grade III (>20 mmHg) in two patients.

### Haemodynamic effects of PLR

**Fig. 1:**
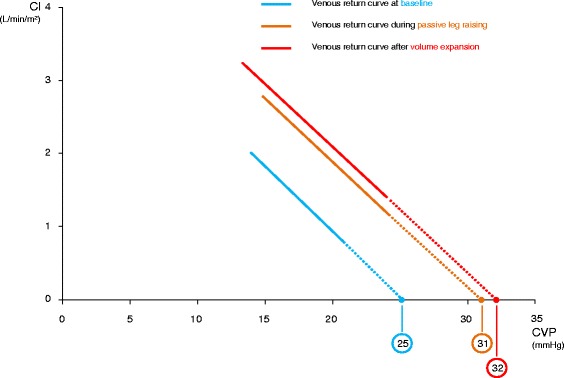
Relationship between cardiac index (*CI*) and central venous pressure (*CVP*). Change at different steps of the study in the case of fluid responsiveness. Values are represented as mean

**Fig. 2:**
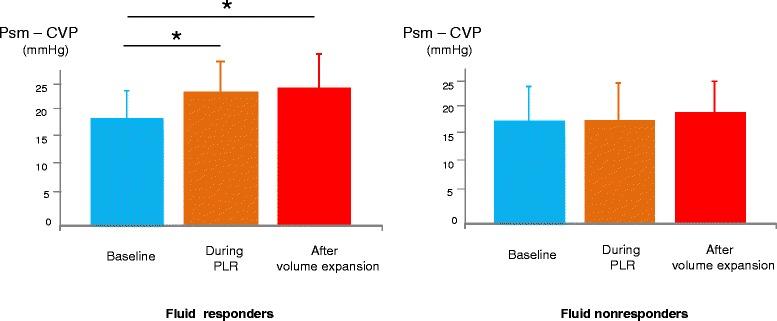
Values of the gradient between mean systemic pressure (*Psm*) and central venous pressure (*CVP*). Change during different steps of the study in fluid-responders and fluid-nonresponders. Values are represented as mean ± SD. *PLR* passive leg raising

In the 15 fluid-responders, PLR increased CI by 17 ± 20 % (Table 2). During PLR, CVP and Psm significantly increased. The Psm-CVP gradient significantly increased by 20 ± 30 % (Figs. 1 and 2, Table 2). Rvr did not change significantly during PLR (Table 2).

**Table 2:** Haemodynamic variables and intra-abdominal pressure at different study times

	Baseline	During passive leg raising	*P* vs. *baseline*	After volume expansion	*P* vs. *baseline*
Heart rate, beats/mm					
Fluid responders	92 ± 16	94 ± 17	0.07	94 ± 15	0.23
Fluid nonresponders	80 ± 21	79 ± 21	0.29	79 ± 19	0.07
Mean arterial pressure, mm Hg					
Fluid responders	67 ± 9	74 ± 10	0.07	76 ± 13	0.04
Fluid nonresponders	79 ± 13	81 ± 20	0.67	83 ± 14	0.13
Mean systemic pressure, mm Hg					
Fluid responders	25 ± 13	31 ± 13	<0.01	32 ± 17	<0.01
Fluid nonresponders	24 ± 10	27 ± 10	<0.01	28 ± 12	<0.01
Cardiac index, L/mm/m^2^					
Fluid responders	2.8 ± 0.9	3.2 ± 0.8	<0.01	3.6 ± 1.1	<0.01
Fluid nonresponders	2.9 ± 1.1	3.0 ± 1.1	0.07	3.0 ± 1.3	0.07
Central venous pressure, mm Hg					
Fluid responders	7 ± 3	9 ± 4	<0.01	9 ± 4	<0.01
Fluid nonresponders	8 ± 4	11 ± 4	<0.01	11 ± 4	<0.01
Inverse of the slope of venous return curve, mmHg.min.m^2^/L					
Fluid responders	6.4 ± 3.7	6.8 ± 3.1	0.54	7.0 ± 5.4	0.35
Fluid nonresponders	5.9 ± 3.8	5.7 ± 3.7	0.75	6.0 ± 3.9	0.84
Resistance to venous return, mmHg.min.m^2^/L					
Fluid responders	6.6 ± 3.5	6.8 ± 4.5	0.66	6.6 ± 4.9	0.93
Fluid nonresponders	5.6 ± 3.5	5.7 ± 3.3	0.65	6.1 ± 4	0.22
Mean systemic pressure - central venous pressure gradient, mmHg					
Fluid responders	19 ± 12	22 ± 12	0,02	23 ± 15	0,02
Fluid nonresponders	16 ± 9	16 ± 9	0.62	17 ± 11	0.33
Intra-abdominal pressure, mm Hg					
Fluid responders	12 ± 6	12 ± 6	1,00	13 ± 6	0.42
Fluid nonresponders	16 ± 5	14 ± 5	0.09	16 ± 6	0.55

Values are expressed as mean ± standard deviation

**Fig. 3:**
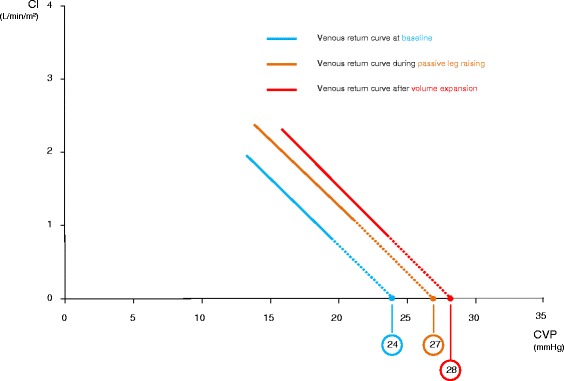
Relationship between cardiac index (*CI*) and central venous pressure (*CVP*). Change at different steps of the study in case of fluid unresponsiveness. Values are represented as mean

In the 15 fluid-nonresponders, CI was unchanged by PLR. During PLR, CVP and Psm increased significantly but the Psm-CVP gradient did not change significantly (Figs. 2 and 3, Table 2).

PLR did not change the intra-abdominal pressure in the whole population (14 ± 6 mmHg before vs. 13 ± 5 mmHg during PLR, *p* = 0.26) or in patients with intra-abdominal hypertension at baseline (17 ± 4 mmHg before vs. 16 ± 4 mmHg during PLR, *p* = 0.14). In the latter group, PLR increased Psm from 22 ± 11 to 27 ± 10 mmHg (*p* <0.01) and did not change Rvr (5.1 ± 2.6 to 5.2 ± 3 mmHg/min/m^2^/mL, *p* = 0.71).

### Haemodynamic effects of fluid infusion

In the 15 fluid-responders, fluid infusion increased CI by 33 ± 20 %. During fluid infusion, CVP and Psm significantly increased. The Psm-CVP gradient significantly increased by 23 ± 27 % (Fig. 2, Table 2). Rvr did not change significantly during fluid infusion (Fig. 1, Table 2).

In the 15 fluid-nonresponders, CVP and Psm increased significantly during fluid infusion but the Psm-CVP gradient did not change significantly (Fig. 2, Table 2). Rvr did not change during fluid infusion in these patients (Fig. 3, Table 2).

Considering the whole population, the PLR-induced changes in CI predicted fluid responsiveness with an area under the receiver operator characteristic (ROC) curve of 0.98 ± 0.03.

## Discussion

This study showed that PLR significantly increased Psm without modifying Rvr. This was also the case in patients with intra-abdominal hypertension. In the case of fluid responsiveness, PLR increased venous return by increasing Psm to a larger extent than CVP. In patients with fluid unresponsiveness, PLR increased Psm but did not change the Psm–CVP gradient and the cardiac output. Fluid infusion induced similar effects on Psm and Rvr.

We estimated the two main determinants of systemic venous return by using heart–lung interactions. By varying the intrathoracic pressure during inspiratory and expiratory holds at two different levels of PEEP, we obtained a series of pairs of measurements of CVP and CI, which were assimilated to right atrial pressure and venous return, respectively. Assimilation of right atrial pressure by CVP is universally accepted. Assimilation of venous return by cardiac output is also accepted in apneic steady-state conditions such as end-expiratory and end-inspiratory occlusion. Initially developed by Maas and co-workers [7], this method has been demonstrated to provide a reliable [7, 12] and precise [12] estimation of Psm. It has been used for demonstrating that norepinephrine increases Psm to a significant extent [8, 13].

PLR is used as a test for predicting fluid responsiveness. The test is based on the assumption that it increases the stressed blood volume by inducing the gravitational transfer of venous blood from the inferior limbs and the splanchnic compartment toward the cardiac cavities [2]. Nevertheless, the effects of PLR on the determinants of venous return have been investigated in one study only [14], but this study did not differentiate the effects of PLR in fluid-responders and in fluid-nonresponders. Moreover, some authors have suggested that the PLR test would not be reliable in case of intra-abdominal hypertension because it would compress the inferior vena cava [4, 15]. Nevertheless, the intra-abdominal pressure has not been measured during PLR in this study [4, 15]. In these regards, our study provides some interesting novel insights into the haemodynamic effects of PLR.

First, PLR induced significant increases in Psm and CVP in the whole population of patients, confirming that it actually represents a powerful preload challenge [2]. These results corroborate those of Keller et al. [14], who reported that PLR increased Psm from 20 to 22 mmHg and CVP from 4 to 6 mmHg. Second, we observed that PLR did not change the intra-abdominal pressure. Moreover, in patients with the highest intra-abdominal pressures, PLR increased the venous return pressure gradient without increasing Rvr. The latter result strongly suggests that intra-abdominal hypertension should not be regarded as a condition in which the PLR test is not valid.

**Fig. 4:**
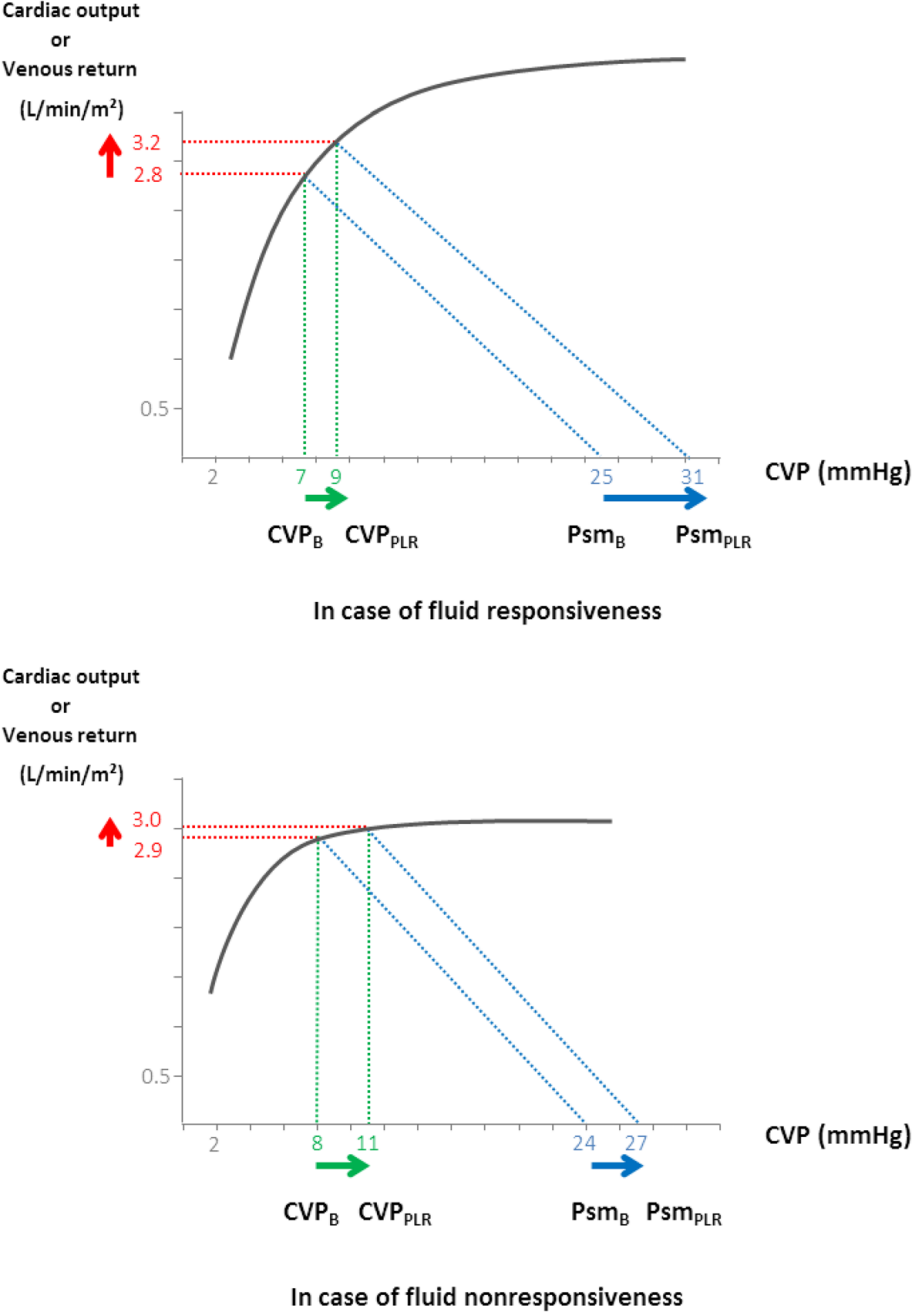
Hypothetical effect of passive leg raising (*PLR*) on the venous return curve in function of the presence of fluid responsiveness. In the case of fluid responsiveness, PLR increased mean systemic pressure (*Psm*) to a larger extent than central venous pressure (*CVP*), because the operating point moved on the steep part of the Frank-Starling curve. This should lead to a significant increase in venous return and cardiac output. By contrast, in the case of fluid unresponsiveness, PLR increased Psm and CVP to a similar extent, because the operating point moved on the flat part of the Frank-Starling curve. This should not lead to a significant increase in venous return and cardiac output. *CVP*_*B*_ CVP at baseline, *CVP*_*PLR*_ CVP during passive leg raising, *Psm*_*B*_ PSM at baseline, *Psm*_*PLR*_ PSM during passive leg raising

One of the major interests of the study was to analyse the effects of PLR depending on the fluid responsiveness status. In patients with fluid responsiveness, both PLR and fluid administration increased the Psm-CVP gradient, which resulted from a larger increase in Psm than in CVP. This increase in the pressure gradient for venous return was associated with an increase in cardiac output and no change in Rvr. Physiologically, Psm depends on vascular compliance and on the volume of venous blood that is submitted to the strain of the venous reservoir walls, i.e., stressed blood volume [16]. As fluid infusion is assumed not to alter vascular compliance, our results suggest that fluid infusion increased Psm and cardiac preload by increasing the stressed blood volume, which confirms the results by Keller and co-workers in a smaller series of patients [14]. Our results suggest that PLR also increased the stressed blood volume. CVP did not increase as much as Psm during PLR and fluid infusion. This was probably related to the fact that in these preload-dependent patients, the heart was working on the steep part of the Frank-Starling curve. Therefore, a rightward shift on the venous return curve induced by the increase in Psm resulted in a smaller increase in CVP (Fig. 4). The increase in the pressure gradient for venous return (Psm-CVP) with no change in Rvr, which occurred during both PLR and fluid infusion was consistent with an increase in venous return. This increase in venous return is attested by the increase in CI measured with an independent method. Interestingly, PLR and fluid infusion did not reduce Rvr in these patients, while a decrease in Rvr due to a reduction in the sympathetic tone could have been expected from an improvement in CI [17]. It is likely that reduction in the sympathetic tone was of too small amplitude to induce significant changes in Rvr. This is in keeping with the observation that heart rate was unchanged by PLR and fluid infusion.

In patients with fluid unresponsiveness, neither PLR nor fluid infusion increased the Psm-CVP gradient (Fig. 4). Indeed, in the case of cardiac preload-independence, the heart operates on the flat part of its Frank-Starling curve so that a rightward shift of the venous return curve (related to the increase in Psm) would result in no increase in cardiac output and in an increase in CVP equal to that of Psm (Fig. 4). In an elegant recent study, using a computerised estimation of Psm before and after fluid infusion, Cecconi et al. reported similar results, with a significant increase in the Psm-CVP gradient in fluid-responders and no change in this gradient in fluid-nonresponders [18]. It is noteworthy that these authors obtained such results with a method estimating Psm and Rvr that was different from ours, which reinforces our observation and interpretation. In the case of fluid unresponsiveness, both the unchanged Psm-CVP difference and the unchanged Rvr during PLR and during fluid infusion suggest that venous return did not change, as confirmed by the absence of change in CI measured with an independent method.

A first potential limitation of this study is that we could not compare the estimation of Psm and Rvr obtained by using heart-lung interactions and the theoretical reference that consists in measuring vascular pressures during cardiac arrest. In our study, Psm was estimated from the extrapolation of the regression lines between CVP and CI pairs of measurement. However, we cannot exclude that this part of the relationship between CVP and CI is non-linear, contrary to what was described by Guyton in animals [1]. Validity of the heart-lung interaction methods that we used is suggested by the observation of high correlation coefficients of the regression lines between the CVP and CI pairs of measurements, which confirms previous results [8]. Second, and as stated above, we did not include patients with severe intra-abdominal hypertension because this syndrome is quite uncommon in medical ICU patients. This prevents us from making conclusions about the possible impairment of venous return in such cases, and deserves further study.

## Conclusion

In conclusion, in preload-dependent patients, PLR and fluid infusion increased Psm and increased CVP to a lesser extent while Rvr remained unchanged. This resulted in an increase in venous return. In preload-independent patients, venous return was unchanged by both PLR and fluid infusion, as CVP and Psm increased to a similar extent.

## Key messages

- PLR increases Psm and CVP, confirming that PLR represents a powerful preload challenge
- Effects of PLR depend on the fluid responsiveness status
- In patients with fluid responsiveness, both PLR and fluid administration increased the Psm-CVP gradient, which resulted from a larger increase in Psm than in CVP. This increase in the pressure gradient for venous return was associated with an increase in cardiac output and no change in Rvr
- PLR and volume expansion have the same effects on venous return in the case of fluid responsiveness or fluid unresponsiveness.

## Abbreviations

CI: cardiac index
CVP: central venous pressure
PEEP: positive end-expiratory pressure
PLR: passive leg raising
Psm: mean systemic pressure
ROC: receiving operating characteristics
Rvr: resistance to venous return
